# Facilely Flexible Imprinted Hemispherical Cavity Array for Effective Plasmonic Coupling as SERS Substrate

**DOI:** 10.3390/nano11123196

**Published:** 2021-11-25

**Authors:** Jihua Xu, Jinmeng Li, Guangxu Guo, Xiaofei Zhao, Zhen Li, Shicai Xu, Chonghui Li, Baoyuan Man, Jing Yu, Chao Zhang

**Affiliations:** 1 Collaborative Innovation Center of Light Manipulations and Applications, Institute of Materials and Clean Energy, School of Physics and Electronics, Shandong Normal University, Jinan 250358, China; xujihua23@163.com (J.X.); jinmeng345@163.com (J.L.); czsdnu@hotmail.com (G.G.); zxfsdnu@126.com (X.Z.); lizhen19910528@163.com (Z.L.); manyuanman_sdnu@163.com (B.M.); 2 Shandong Key Laboratory of Biophysics, Institute of Biophysics, Dezhou University, Dezhou 253023, China; shicaixu@dzu.edu.cn (S.X.); chonghuili163@163.com (C.L.)

**Keywords:** surface-enhanced Raman spectroscopy, localized surface plasmon resonance, flexible SERS substrate, cavity

## Abstract

The focusing field effect excited by the cavity mode has a positive coupling effect with the metal localized surface plasmon resonance (LSPR) effect, which can stimulate a stronger local electromagnetic field. Therefore, we combined the self-organizing process for component and array manufacturing with imprinting technology to construct a cheap and reproducible flexible polyvinyl alcohol (PVA) nanocavity array decorating with the silver nanoparticles (Ag NPs). The distribution of the local electromagnetic field was simulated theoretically, and the surface-enhanced Raman scattering (SERS) performance of the substrate was evaluated experimentally. The substrate shows excellent mechanical stability in bending experiments. It was proved theoretically and experimentally that the substrate still provides a stable signal when the excited light is incident from different angles. This flexible substrate can achieve low-cost, highly sensitive, uniform and conducive SERS detection, especially in situ detection, which shows a promising application prospect in food safety and biomedicine.

## 1. Introduction

The Raman spectrum has been widely used in qualitative and quantitative analysis as a nondestructive analysis tool, which can provide molecular vibration information [1]. However, the weak signal due to the low Raman cross-section area greatly hinders the development of this technology [2,3]. Surface-enhanced Raman scattering (SERS) has been widely used in food and environmental safety, early disease diagnosis, drug detection, in situ monitoring of biological environment and chemical reactions, which can efficiently enhance the Raman signal [4,5,6,7,8]. As we all know, the amplification of the Raman signal is usually realized by an electromagnetic mechanism that is mainly depends on the localized surface plasmon resonance (LSPR) effect whereby collective oscillation of the metal’s conduction band electrons is excited upon light excitation [9]. It is reported that the electromagnetic field intensity is inversely proportional to the distance parameter, which means that the effective SERS signal is generated on the premise that the molecule is located in the confined area around the plasmonic material [10]. Various types of SERS substrate structures ranging from nanoparticle (NP) aggregates [11,12,13,14,15], multidimensional structure [16,17,18,19,20] and composite substrate [21,22,23,24,25,26,27,28,29] were prepared to explore the better enhancement factor (EF) under various conditions. Among them, the cavity system has been paid more and more attention because of its unique light trapping characteristics [30,31,32,33,34,35,36,37,38]. The combination of surface and cavity plasmonic modes can provide an effective way for electromagnetic field enhancement. For instance, alumina nanocavity arrays were prepared by drying and decomposing the aqueous solution of Al (NO_3_)_3_ spun on the monolayers of PS spheres [39], which provided the distribution of hotspots in three-dimensional space. Compared with uncomplicated structures (such as nanoparticles and films), the light reflection caused by gradient refractive index and multiple internal scattering can be effectively suppressed in metal nanocavities [40]. Inspired by the equivalence of light propagation between media containing gradients in optical properties and warped geometries of space-time, Peng Mao et al. designed and proved that warped spaces can be used to introduce extremely localized energy [41]. More recently, Zewen Zuo et al. prepared a polydimethylsiloxane-supported Ag nanocone array covered by gold nanoparticles (Au NP), where multiple types of plasmonic coupling occur in this multi-focus field mode, resulting in significantly enhanced electromagnetic fields and large hot spots area. Generally, ultra-high sensitivity is the basic performance of a superior SERS sensor, but signal stability, uniformity and low production cost in the real-work application are also crucial issues to be investigated [42,43].

Most of the substrate templates used to fabricate cavity structures relied too much on expensive and energy-consuming precision machining processes, such as electron beam lithography and focused ion beam pattern lithography, which greatly limits the practical application of high-performance and reliable SERS substrates. Based on this strategy, we combined the self-assembly technology with the imprinting process to prepare a periodic hemispherical cavity array structure, which can be used as a highly sensitive sensor for transmitting stable, uniform and repeatable signals. Here, we chose PVA as the template layer and the PS microsphere monolayer self-assembled in periodical arrangements as the object to be imprinted to obtain a flexible imprinted hemispherical cavity array. Then, the silver nanoparticles are sputtered in the hemispherical cavity to synergize the metal surface plasmon resonance (LSPR) with the cavity mode, which further enhances the Raman signal. With the aim of calculating the enhancement effect of this substrate, we detected different concentrations of rhodamine 6G (R6G), Crystal Viole (CV) and Malachite green (MG); their Raman signals were enhanced to varying degrees, accompanied by good uniformity. The geometry of metal nanostructures can affect the plasmon resonance effect to a great extent; thus, we compared the different roles of silver film and silver nanoparticles in the cavity. The effects of Ag film and Ag nanoparticles on the strength of hot spots were compared by finite-different time-domain (FDTD) simulation. When silver nanoparticles are orderly attached to the inner wall of the hemispherical cavity, more hot spots are generated between the adjacent nanoparticles. The three-dimensional distribution of hot spots in Ag NP-cavity simulated by COMSOL confirmed this point. In addition, the stability of the substrate was also tested. As the results show, the flexible substrate can be used for quantitative and qualitative analysis and has a wonderful application prospect for reliable in situ detection.

## 2. Materials and Methods

### 2.1. Chemicals and Materials

Acetone (CH_3_COCH_3_), Ethanol (C_2_H_6_O), Sodium dodecyl sulfate (SDS, C_12_H_25_NaO_4_S) and Toluene (C_7_H_8_) were purchased from Sinopharm Chemical Reagent Co., Ltd. (Shanghai, China). Monodisperse polystyrene (PS) sphere suspensions (5 wt% in water) with diameters of 500 nm were purchased from Maxinne dareen (China). Single-sided polished silicon wafers were purchased from Lijing Technology Co., Ltd. (Shenzhen, China). PVA 1788 (87.0–89.0% alcoholysis degree) and malachite green (MG, MW:463) were purchased from Shanghai Aladdin Biochemical Technology Co., Ltd. (Shanghai, China). Rhodamine 6G (R6G, MW:479) was purchased from Meilun Biotechnology Co., Ltd. (Dalian, China). Crystal violet (CV, MW:407) was purchased from Yuanye Biotechnology Co., Ltd. (Shanghai, China).

### 2.2. Preparation of Tightly Packed Polystyrene Sphere Monolayer

PS microsphere arrays were prepared by gas-water interface self-assembly technology: The cleaned slides were firstly treated by oxygen plasma for 120 s to obtain a highly hydrophilic surface. Subsequently, after mixing ethanol and PS microspheres at the ratio of 2:1 and ultrasonic treatment of 15 min, a small amount of the mixture was dripped on the slide, covering the entire upper surface of the slide. Then, it was left at room temperature for an hour until a large area of the continuous molecular layer of PS microspheres was formed on the surface of the slide. Sodium dodecyl sulfate with a concentration of 2 wt% was prepared as a surfactant drop on the surface of deionized water (DI) to change the surface tension of the DI and drive the nanospheres into a hexagonal and tightly packed monolayer [44]. The PS microsphere monolayer on the glass slide was detached from the deionized water surface in the beaker, and then the molecular film was obtained from the silicon wafer, which was also treated by oxygen plasma; thus, the self-assembly monolayer of the PS microsphere was transferred to the silicon wafer.

### 2.3. Preparation of Ag NPs/PVA Nano-Bowl Cavity Array Substrate

In order to make the PS microspheres firmly adhere to the silicon wafer surface, we heated the PS spherical monolayer on the silicon wafer surface to 20 min at 90 °C PVA aqueous solutions with different concentrations were prepared by stirring the mixture of polyvinyl alcohol powder and deionized water at 80 °C for 6 h, and then spin-coated on the monolayer of PS spheres by a homogenizing machine and dried at 60 °C for 40 min to obtain sandwich structure on silicon wafers. With the transparency of PVA in mind, we stuck a tiny label film that is chemically stable and has no effect on the substrate to the corner of the sample to distinguish between the positive and negative sides of the substrate. Before completely soaking and etching the PS microspheres in toluene solution, the silicon wafers were peeled off to obtain the structure of a single-layer PS microsphere array covered with PVA. After soaking in toluene solution for 40 h, the PVA flexible substrate with sunken bowl-shaped cavity array (PC) was fabricated. Silver nanoparticles were Sputtered on the PVA bowl cavity structure by the Denton Vacuum DESKTOP PRO turbo pumped confocal sputter deposition system, and the size of Ag NPs was changed by setting different times. The preparing technology was completed when the sample was coated with an ultra-thin gold film (3 nm) to slow down the oxidation reaction, the Ag NPs/PVA bowl-shaped cavity array composition was obtained.

### 2.4. Characterization

The morphologies and compositions of the prepared samples were characterized by scanning electron microscopy (SEM, Zeiss Gemini Ultra-55, Jena, Germany) equipped with energy-dispersive X-ray spectroscopy. SERS spectra were measured using the Horiba HR Evolution 800 Raman spectrometer (Kyoto, Japan). Raman measurement conditions: laser power = 0.48 mW; diffraction grid = 600 g/nm; objective lens = 50×; laser wavelength = 532 nm. All the detected molecules were prepared by dissolving them into ethanol solution.

### 2.5. Theoretical Simulation

The electromagnetic field distributions of Ag NPs/PVA nano-bowl cavity (Ag NP-on-PC) and Ag film-on-PC structures were first simulated by FDTD, and the *x*-*z* cross-sectional views were compared. In order to visualize the distribution of hot spots in the 3D nanocavity, we then used commercial COMSOL Multiphysics software to simulate the distribution of silver nanoparticles in the PVA cavity. To control the uniqueness variables, in all models, the diameters of silver nanoparticles and hemispherical cavities were set to 35 nm and 500 nm, and the nano-gap between the particles was set to 3 nm. The polarization direction was set along the *x*-axis. In all the directions, the absorption boundary condition was the perfect matching layer (PML). The incident light with the wavelength of 532 nm was used and incident along the *z*-direction. The corresponding refractive index of PVA was set as 1.4835 and the dielectric functions of Ag were taken from Palik. The refractive index of the surrounding medium was 1.0 for air.

## 3. Results

### 3.1. Morphologies of PVA Nanocavity Arrays and the Effect of the Concentration of PVA

**Figure 1:**
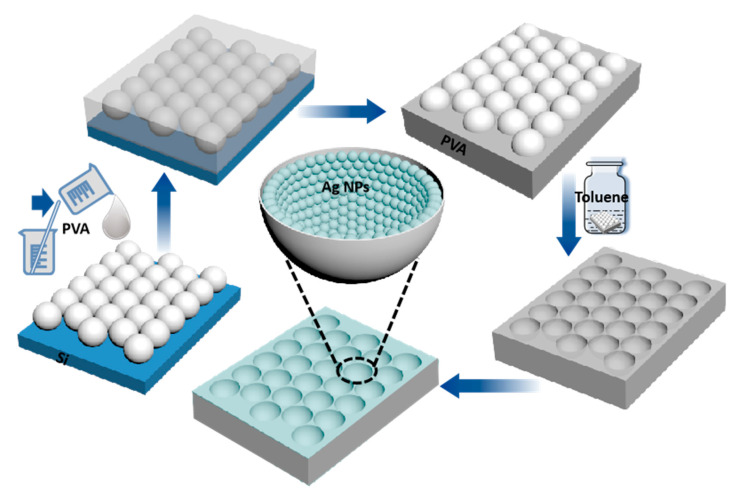
The fabrication process of hierarchical Ag NPs/PVA bowl-shaped cavity array nanostructure.

**Figure 2:**
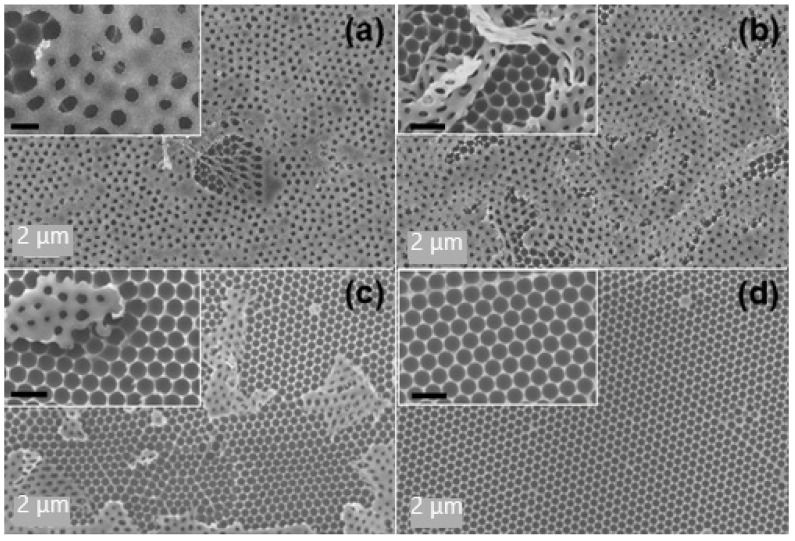
Morphology of the fabricated sunken bowl-shaped microcavity arrays, which were obtained by preparing a PVA solution with a concentration of (**a**) 10 wt% (PC-10), (**b**) 15 wt% (PC-15), (**c**) 20 wt% (PC-20) and (**d**) 25 wt% (PC-25), respectively.

The preparation process of the 3D flexible Ag NPs/PVA bowl-shaped cavity array substrate is illustrated schematically in Figure 1. More information about the synthesis process and SERS spectrum measurement is shown in the Materials and Methods. During the preparation of the PVA flexible nano-bowl array template, we found that different morphologies could be obtained by covering different concentrations of polyvinyl alcohol on the monolayer PS microsphere array. The SEM images of these structures are shown for comparison. The morphology dependence of the concentration of PVA solution can be understood from the fluidity before it is fixed to form hemispherical cavities. When the concentration is 10 wt% (Figure 2a), the extremely fluid PVA permeates every gap between the microspheres, leaving a double-layer solidified reticular structure of PVA after the microspheres are completely corroded. When the concentration of PVA increases to 15 wt%, the PVA solution will not flow to the bottom of some PS microspheres, and the monolayer nano-bowl structure begins to appear. As the concentration increases, the fluidity of the PVA solution weakens, the area of double-layer reticular decreases, and the substrate gradually tends to be uniform. When the concentration of PVA reaches 25 wt%, we obtain a large area of uniform and tightly arranged bowl-shaped cavity array structure as shown in Figure 2d. The efficient and intuitive views illustrate the effect of the concentration of PVA solution on the morphology.

**Figure 3:**
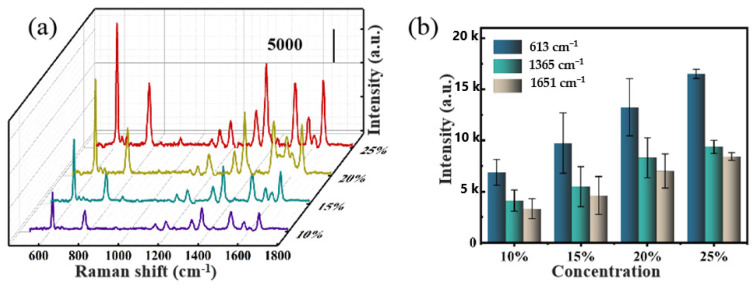
(**a**) The Raman spectra of R6G molecules were detected on these four different structures, which were obtained with the PVA concentration of 10 wt% (Ag-PC-10), 15 wt% (Ag-PC-15), 20 wt% (Ag-PC-20) and 25 wt% (Ag-PC-25). The Raman characteristic peak intensities of R6G are shown on (**b**), respectively. It is calculated that RSD = 18.14%, 30.31%, 21.08%, 5.9%, correspondingly.

In order to endow SERS activity, silver nanoparticles were sputtered on the surface of the obtained PVA flexible nano-bowl array template. Subsequently, we separately detected the R6G molecule with a concentration of 10^−6^ M on these different structures, and the results are shown in Figure 3. Apparently, the double-decker covered with a reticular composition exhibits relatively poor SERS activity. As can be seen in the illustration in Figure 2a–c, the diameter of the upper meshwork is smaller than the underlying hemispherical cavity so that the silver electron beams are blocked and composed of nanoparticles on the upper meshwork. The number of Ag nanoparticles attached to the inner wall of the hemispherical cavity is much lower; thereupon, the Ag NP-cavity system cannot be formed, which weakens the coupling effect between metal plasma and intracavitary-focusing field, the Raman performance was greatly reduced. In contrast, the remarkably strongest Raman signal was detected on the Ag-PC-25 substrate, indicating that the pure hemispherical cavity structure is more conducive to coupling.

### 3.2. SERS Performance and the Dependence of the Ag NPs/PVA Nanocavity Array

**Figure 4:**
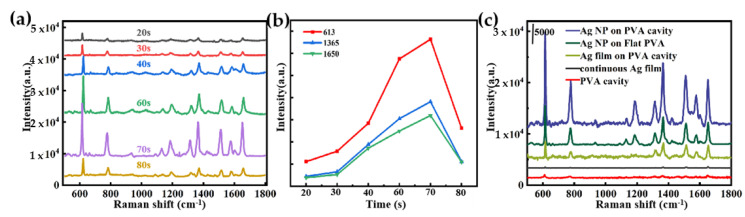
(**a**) SERS spectra of R6G (10^−6^ M) over substrates with different sputtering times were detected, and (**b**) the intensity of the corresponding characteristic peaks were shown. (**c**) SERS spectra of R6G (10^−2^ M) over PVA cavity, and the spectra of R6G (10^−6^ M) over Ag NP-on-PC, Ag NP-on Flat PVA(FP), Ag film-on-PC and continuous Ag film substrates.

Figure 4a presents the Raman spectra of the 10^−6^ M R6G alcoholic solution detected on fabricated Ag NPs/PVA nanocavity arrays, which were obtained by sputtering silver with different duration, where the change in the Raman signal intensity with increasing the deposition time from 20 s to 80 s is exhibited. Evidently, the optimal enhancement effect on the Raman signal can be achieved when the sputtering time is 70 s. The intensities of the Raman peaks at 613, 1365 and 1651 cm^−1^ plotted as functions of the sputtering time are shown in Figure 4b, from which we can observe that the Raman intensities increase with Ag sputtering time increasing until 70 s, and then decrease when the time continues to increase. The SERS activity dependence of the sputtering time can be understood from the coupling strength of the cavity mode with the metal particle plasma. In the process of deposition, 3D-coated silver nanoparticles instead of continuous films are produced in the early stage due to the limited wettability of the substrate [45]. When the sputtering time is not long enough, the small size of the nanoparticles not only leads to the weak LSPR effect but also weakens the focusing field effect because the cavity film is not formed. When the sputtering time is appropriate, a sufficient amount of LSPR is generated between the silver nanoparticles, and all the particles attached to the bowl-shaped inner wall form a focusing field. There is a strong coupling between the two modes, and with the enhancement of the coupling, a stronger hot spot is produced. However, when the sputtering continues after the “best time”, the gap decreases gradually, the hot spot decreases, and the coupling effect weakens. For a homogeneous material, it was proved that the Raman properties of nanoparticles on planar substrates are much worse than those on warped surfaces because of their uniform refractive index and electromagnetic field, although other variables are consistent [46,47]. To that end, we collected the SERS spectra of R6G over PVA cavity, Ag NP-on-PC, Ag film-on-PC and continuous Ag film substrates in addition. As illustrated in Figure 4c, all the characteristic Raman peaks of R6G can be detected in these four cases, in which the enhancement effect of the PVA cavity was measured with R6G at a concentration of 10^−2^ M, and R6G at the concentration of 10^−6^ M is tested for the other substrates. Obviously, it can be seen the enhancement effect of Ag NP-on-PC structure is much stronger than that of that on FP, which is attributed to the cavity mode. It is expected that the higher signal strength exhibited on Ag NP-on-PC, whether compared to Ag film-on-PC or nudity PVA cavity, provides further evidence of the high-density hot spots generated because of the synergy.

**Figure 5:**
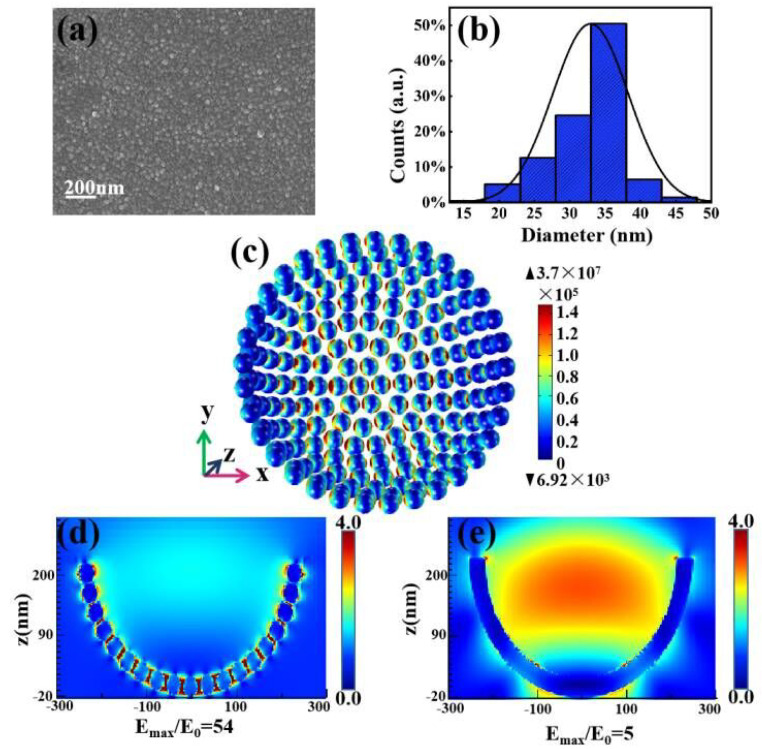
(**a**) SEM images of Ag NPs sputtered on the flat PVA film (Ag NP-on-FP) under the same conditions with Ag NP-on-PC. (**b**) The distribution of particle diameter is calculated. (**c**) Simulated electric field distribution of Ag NPs in the inner wall of the cavity and the *x*-*z* plane electric field distribution of (**d**) Ag NP-on-PC and (**e**) Ag film-on-PC.

We implement 3D simulations of hot spots among nanoparticles in the cavity by using COMSOL. Before establishing the model, we calculated a statistical analysis of the diameter distribution of Ag NPs based on SEM images of Ag NP-on-FP obtained in the same experimental environment (Figure 5a), acquiring a logarithmic normal distribution with a mean value of 35 nm and a standard deviation of 5 nm, as shown as in Figure 5b. In Figure 5c, it can be seen that the hot spots excited by the LSPR effect are 3D-distributed between the adjacent particles. In order to further demonstrate the influence of silver nanoparticles and silver film on the focusing field effect, we simulated the Ag NP-on-PC and Ag film-on-PC structures. The incident light is concentrated and forms a strong focusing field in the hemispherical cavity under 532 nm light excitation. The local electric field distributions at x-z cross-section of these two structures were shown in Figure 5d,e, where it can be observed that the Ag NP-cavity can produce more local electromagnetic fields (hot spots) distributed in the narrow gaps between the silver nanoparticles compared with the Ag film-cavity. In addition, the weak reflection ability caused by its discontinuity produces a weak focus field similar to the latter. On the other hand, the silver film cavity can excite a stronger focusing field mode; even so, its overall enhancement effect is not comparable to that of silver nanoparticles. As we know, the metal nanoparticles themselves can produce a higher local electric field, and there is coupling between the particles, resulting in more hot spots. Because the focusing field has a larger electromagnetic (EM) energy density than the incident plane wave, the plasmon resonance excited by the focusing field makes the local field of the nanoparticles stronger, thus strengthening the coupling between the particles. As a result, a further enhanced electric field is generated in the gap between the adjacent particles, which leads to stronger hot spots in the focus field of the Ag NP-cavity, although the focusing field is relatively weak. It was proved that the synergistic effect of plasmon resonance of metal nanoparticles and cavity mode is particularly effective for SERS.

### 3.3. Optimized Ag NPs/PVA Nanocavity and SERS Performance

**Figure 6:**
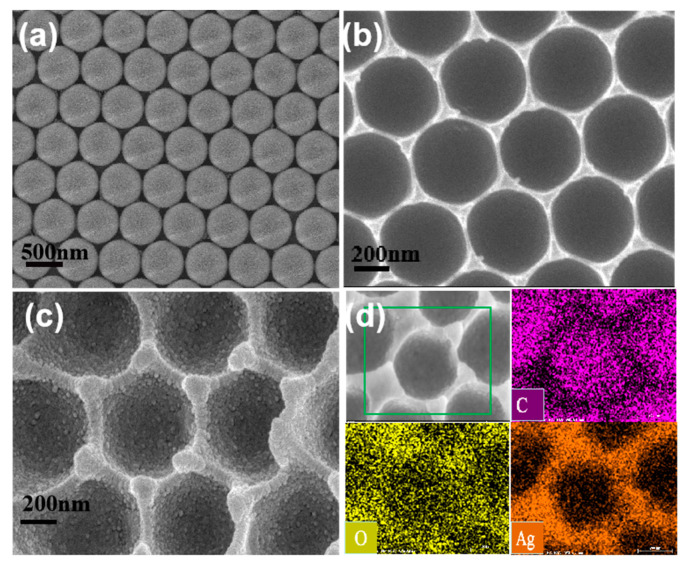
Morphology characterization from PS microsphere array (**a**), hemispherical cavity array (**b**), Ag NPs/PVA cavity composite substrate (**c**), respectively. (**d**) EDS elemental maps from C, O and Ag on (**c**).

The morphology and structure of the PS microsphere array (Figure 6a), PVA cavity array (Figure 6b) and hemispherical cavity array assembled by Ag nanoparticles (Figure 6c) were characterized by scanning electron microscope (SEM). In Figure 6b, it can be seen that the cavity array was composed of hexagonally arranged hemispherical voids, which replicate the features of the PS microsphere array well. Figure 6c exhibits the morphology of intact substrate, where the hexagonal arrangement and the closely stacked concave array structure were observed in the low power scanning electron microscope images, which was uniformly covered with silver nanoparticles, and the average size was about 30 nm. EDS elemental mappings (Figure 6d) of local samples showed that carbon (purple), oxygen (yellow) and silver (orange) elements were uniformly distributed on the tested samples. The large-scale highly ordered inverse PS hemispherical monolayer template ensures the successful preparation of the hemispherical cavity array and further ensures the high uniformity of SERS spectra at different positions on the whole substrate. This simple method makes the silver hemispherical cavity array assembled by different batches of silver nanoparticles have high structural repeatability, thus ensuring the high repeatability of SERS spectra. In addition, we also measured the UV–vis spectra of Ag NPs/PVA nanocavity and pure PVA film (Figure S1a,b), and it can be seen that composite substrate maintains a higher absorption of light with the band from 200 to 500 nm, which is related to its low reflectivity in this band as shown in Figure S1b.

**Figure 7:**
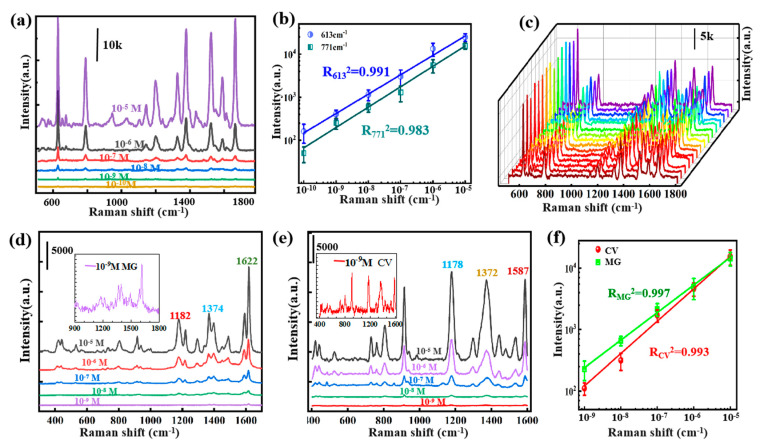
(**a**) SERS-normalized spectra of R6G on Ag NPs/PVA cavity composite structure at varying concentrations from 10^−5^ to 10^−10^ M. (**b**) Linear relationships: Raman intensities at 613 and 771 cm^−1^ as a function of the concentrations of R6G molecules on Ag NPs/PVA cavity composite structure. (**c**) The waterfall map consists of Raman spectra obtained by detecting target molecules with different batches of samples (Ag NPs/PVA cavity composite structure). (**d**,**e**) SERS spectra of MG and CV at varying concentrations from 10^−5^ to 10^−9^ M detected on the Ag NPs/PVA composite substrate. Insets in (**d**,**e**) are the enlarged SERS spectrum of 10^−9^ M MG and CV, respectively. (**f**) Plots of the logarithmic integrated intensities of the Raman peaks at 1372 cm^−1^ for CV and 1622 cm^−1^ for MG as functions of the logarithmic concentration, and the solid lines are the linear fittings of the data.

In order to evaluate the Raman properties of the Ag NPs/PVA cavity composite structure, R6G, MG and CV alcoholic solution were prepared to be detected as probe molecules, and the SERS spectra of them are shown in Figure 7a,d,e, respectively. Apparently, the intensity of the Raman signal lessens gradually with the decrease in the concentration of probe molecules. Figure 7a shows the SERS spectra of R6G alcoholic solution with concentrations ranging from 10^−5^ to 10^−10^ M. It can be observed that distinguishable peaks are demonstrated even at diluted concentrations of 10^−10^ M, and the limit of detection (LOD) of this flexible substrate is obtained. In order to quantize the SERS performances, the enhanced factor (*EF*) was evaluated using the formula [48]:

$$EF=\frac{I_{SERS}/N_{SERS}}{I_{RS}/N_{RS}}$$

where *I_SERS_* and *I_RS_* represent the intensity of SERS spectra and normal Raman and *N_SERS_* and *N_RS_* refer to the average number of molecules within the laser spot excited by SERS and normal Raman, respectively. Here, the value of *N_RS_/N_SERS_* was estimated with the ratio of the respective molecule concentrations. The peak strength of 10^−2^ M R6G at 613 cm ^−1^ on PVA is 232, and the peak intensity of 10^−10^ M R6G on Ag NPs/PVA cavity composite structure is 637 (Figure S2). As a result, the average EF is 2.75 × 10^8^, which proves that this substrate has high SERS activity. In order to examine the ability of quantitative detection, the experimental values were logarithmically plotted to evaluate the correlation between concentration and relative intensity at 613 and 771 cm^−1^, and then the linear fitting curve (two solid lines) with a high coefficient of determination (R^2^_613_ = 0.991, R^2^_771_ = 0.983) was obtained, as shown in Figure 7b. Repeatability and universality are two indispensable indicators to measure the practical application of the SERS sensor.

Figure 7c presents the Raman spectra of different batches of R6G with the concentration as 10^−6^ M confirming the reliability of the substrate to generate reproducible SERS signals. Then MG and CV molecules with concentrations of 10^−5^ to 10^−9^ M were also detected; the curves in Figure 7d,e correspond to their Raman spectra in turn. For the spectrum measured at the concentration of 10^−9^ M, the main characteristic peaks of MG and CV can still be clearly distinguished, as shown in the illustration. Similarly, the intensity values of the corresponding characteristic peaks of the two substances at each concentration are linearly fitted, and it is calculated that R^2^_MG_ = 0.997 (green line) and R^2^_CV_ = 0.993 (red line) as shown in Figure 7f. The results of these linear analyses well prove the quantitative analysis ability of the substrate. In addition, three kinds of probe molecules can be detected quantitatively, which proves that the Ag NPs/PVA composite structure is universal in the practical application of Raman detection.

**Figure 8:**
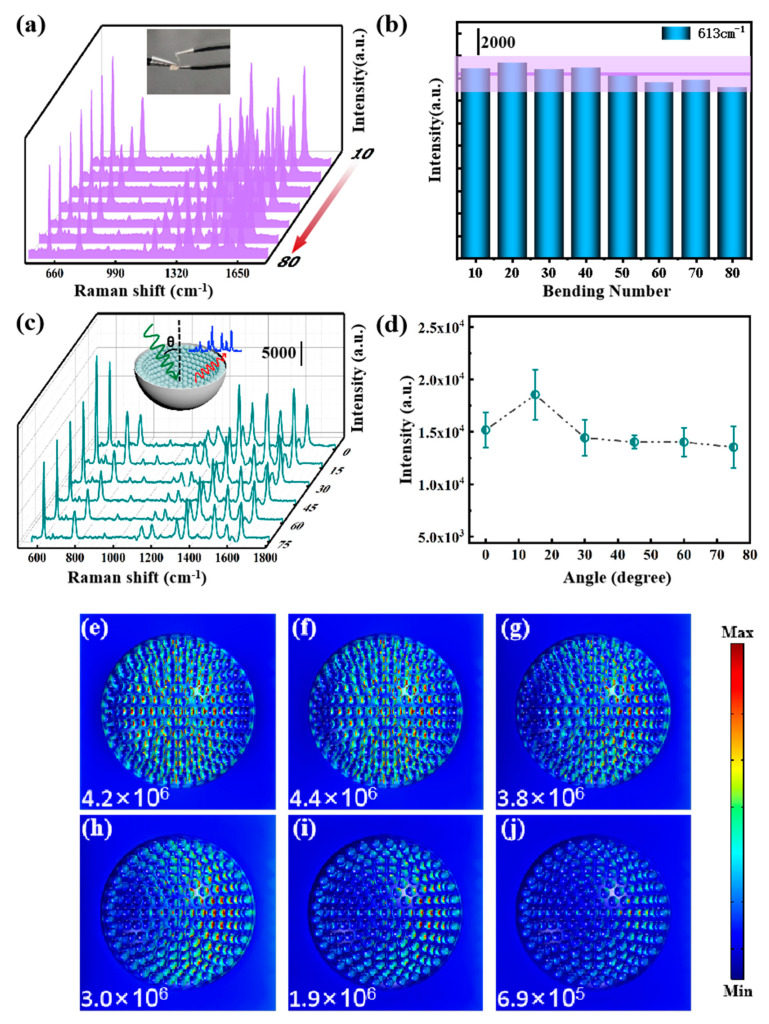
(**a**) Real-time SERS spectra of R6G on Ag NPs/PVA cavity during the bending experiment, and the inset shows the implementation process of bending experiment. (**b**) The corresponding histogram of intensity distribution of the peak at 613 cm^−1^ (the average intensity is marked with a purple line, and the purple zone represents the ±10% fluctuation). (**c**) SERS spectra of R6G on Ag NPs/PVA cavity with laser incidence from different angles (0°, 15°, 30°, 45°, 60°, 75°). The schematic illustration is shown in the inset. (**d**) The corresponding histogram of the intensity of the peak at 613 cm^−1^. (**e**–**j**) Electric field distribution by COMSOL simulation of an optical incidence angle from 0° to 75° for every 5°.

By taking practical application into account, high sensitivity is only one of the critical aspects that the substrate should provide, and stability signal is also very important. To further examine the application performance of Ag NPs/PVA cavity composite structure, the mechanical stability of the thin film was tested firstly. We bent the substrate repeatedly and caught the real-time response after the substrate was restored to steady state, and the SERS spectra were obtained, as shown in Figure 8a. Figure 8b presents the intensity at 613 cm^−1^ of these Raman spectra. The changing trend of Raman signals with the increase in bending times from these histograms can be more intuitively observed. As we can see, the Raman signal detected has a slight downward trend with the increase in bending times because the micro-deformation of the bowl-shaped cavity changed the gap between the silver nanoparticles attached to the inner wall of the cavity driven by the external force and the hot spot changed accordingly. The “*n*” was defined as the ratio of the reduced peak intensity (Δ*E*) to its original intensity (*E*), *n*_80_ = 7.02%, which means that even under the interference of repeated 80 times of high-frequency bending, a slight decrease occurred in peak intensity, which will have a negligible impact on quantitative detection. Furthermore, R6G on Ag NPs/PVA composite structure was detected with laser incident from different angles to investigate the optical stability of the substrate. Generally speaking, strong coupling occurs only when the polarization direction of the excited light is perpendicular to the particle-film interface in the particle-on-film system [49]. As shown in Figure 8c,d, therefore, there is a slight enhancement when the incident angle is 15°. The Raman signal is basically maintained at a stable level with slight fluctuations. In order to further verify this conclusion, we carried out a theoretical simulation, as shown in Figure 8e–j. It can be seen from the simulation results that, as the detected Raman signal shows, the intensity of hot spots changes slightly in a small category. Especially when the incident angle is less than or equal to 60 degrees, the hot spot intensity remains in order of magnitude. Thus far, the mechanical stability and optical stability of the flexible substrate have been verified, which demonstrates that Ag/PVA cavity substrate is a promising candidate for practical curved surface SERS detection.

## 4. Conclusions

To summarize, we developed a flexible 3D Ag NPs/PVA cavity array composite structure that produces dense hot spots through the coupling effect between the focusing field of the incident light and the plasmon effect of Ag NPs in hemispherical cavities. It was shown that the corresponding morphologies of cavity layer structures with various concentrations of PVA solution. The effect of different sputtering times on the Raman properties of the substrate was discussed. Through theoretical simulation, it was concluded that under the excitation of the same cavity mode, Ag NP-cavity could produce a stronger coupling effect than intact Ag film-cavity, which is consistent with the experimental results. Using R6G, CV and MG as probe molecules for Raman detection, the composite substrate shows excellent enhancement effect, outstanding uniformity. Moreover, the signal stability of the substrate is evaluated by changing the incident angle of the laser and the bending times of the film. These results support that it is expected to be used for quantitative and qualitative SERS analysis in environmental monitoring and food safety.

## Data Availability

Data are available within this manuscript and the Supplementary Material.

## Supplementary Materials

The following are available online at https://www.mdpi.com/article/10.3390/nano11123196/s1, Figure S1: UV–vis spectra of Ag NPs/PVA nanocavity (a) and PVA film (b) in a wavelength range between 200 nm and 800 nm. Figure S2: The Raman spectra of 10^−10^ M R6G molecules were detected on Ag NP-on PC and 10^−2^ M R6G molecules on PVA.
