# Supplementary material for: Facilely Flexible Imprinted Hemispherical Cavity Array for Effective Plasmonic Coupling as SERS Substrate
Source: Nanomaterials (Basel). 2021 Nov 25;11(12):3196. doi: 10.3390/nano11123196 (PMC8705896; doi:10.3390/nano11123196)
Supplement: Supplementary file 1 [file nanomaterials-11-03196-s001.zip › nanomaterials-1479714-supplementary.pdf]

nanomaterials

Supplementary Materials

Facilely Flexible Imprinted Hemispherical Cavity Array for Effective Plasmonic Coupling as SERS Substrate

Jihua Xu [1], Jinmeng Li [1] , Guangxu Guo[1], Xiaofei Zhao[1], Zhen Li[1], Shicai Xu[2], Chonghui Li[2], Baoyuan Man[1], Jing Yu[1,*] and Chao Zhang [1,*]

1 Collaborative Innovation Center of Light Manipulations and Applications, Institute of Materials and Clean Energy, School of Physics and Electronics, Shandong Normal University, Jinan 250358, China; xujihua23@163.com (J.X.); jinmeng345@163.com (J.L.); czsdnu@hotmail.com (G.G.); zxfsdnu@126.com (X.Z.); lizhen19910528@163.com (Z.L.); manyuanman_sdnu@163.com (B.M.)
2 Shandong Key Laboratory of Biophysics, Institute of Biophysics, Dezhou University, Dezhou 253023, China; shicaixu@dzu.edu.cn (S.X.); chonghuili163@163.com (C.L.)
* Correspondence: yujing1608@126.com (J.Y.); czsdnu@126.com (C.Z.)

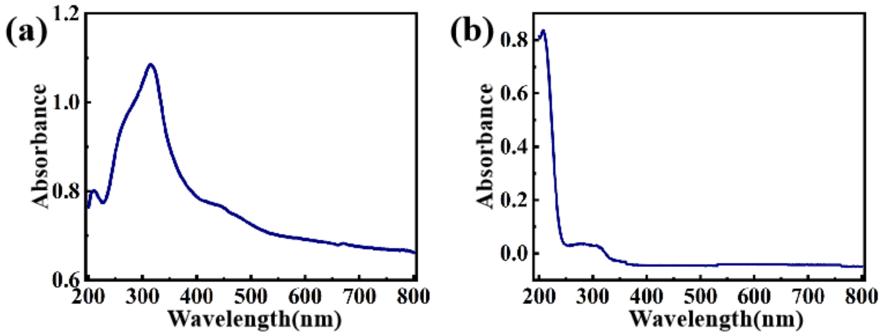

Figure S1. UV–vis spectra of Ag NPs/PVA nanocavity (**a**) and PVA film (**b**) in a wavelength range between 200 nm and 800 nm.

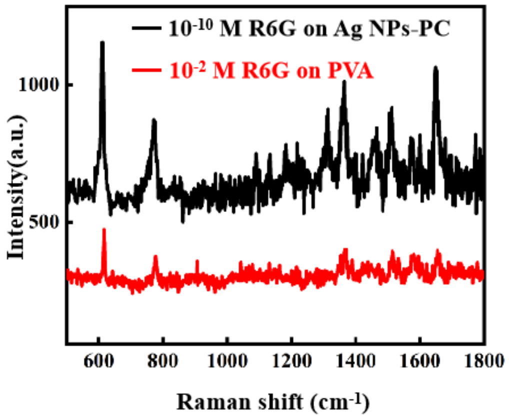

Figure S2. The Raman spectra of 10^{-10} M R6G molecules were detected on Ag NP-on PC and 10^{-2} M R6G molcules on PVA.
